# A 10-Year Single-Center Study of the Clinical Characteristics of Optic Neuritis-Related NMOSD, MS, and Double Seronegative Optic Neuritis, Together with Factors Predicting Visual Outcomes

**DOI:** 10.3390/vision7010016

**Published:** 2023-02-28

**Authors:** Parinee Kemchoknatee, Chotika Singhakul, Niracha Arjkongharn, Methaphon Chainakul, Duanghathai Tangon, Thansit Srisombut

**Affiliations:** 1 Department of Ophthalmology, Rajavithi Hospital, Rangsit University, 2 Phaya Thai Rd., Thung Phaya Thai, Ratchathewi, Bangkok 10400, Thailand; 2 Faculty of Medicine Rajavithi Hospital, Rangsit University, 2 Phaya Thai Rd., Thung Phaya Thai, Ratchathewi, Bangkok 10400, Thailand

**Keywords:** optic neuritis, neuromyelitis optica spectrum disorder, multiple sclerosis, visual acuity

## Abstract

The clinical characteristics of three types of optic neuritis (double seronegative optic neuritis; DN-ON, Neuromyelitis optica spectrum disorder-related optic neuritis; NMOSD-ON, and multiple sclerosis-related optic neuritis; MS-ON) were examined in order to identify factors that may affect good visual recovery in Thai patients. The study included patients diagnosed with three types of optic neuritis at Rajavithi Hospital between 2011 and 2020. Visual acuity at the end of 12 months was used as the treatment outcome. Multiple logistic regression analysis was used to evaluate potential predictors of good visual recovery. Of the 76 patients, 61 had optic neuritis, with DN-ON as the most common subtype (52.6%). MS-ON patients were significantly younger (28.3 ± 6.6 years, *p* = 0.002) and there was a female predominance in all subgroups (*p* = 0.076). NMOSD-ON patients had a significantly higher proportion of poor baseline VA (*p* < 0.001). None of the NMOSD-ON patients achieved 0.3 logMAR visual recovery in the 12-month period (*p* = 0.022). A delay in treatment with intravenous methylprednisolone (IVMP) for more than 7 days increased the risk of failure to gain 0.3 logMAR visual recovery by five times (OR 5.29, 95% CI 1.359–20.616, *p* = 0.016), with NMOSD-ON as the strongest predictor (OR 10.47, 95% CI; 1.095–99.993, *p* = 0.041). Early treatment with intravenous methylprednisolone may be important for achieving at least 0.3 logMAR visual recovery in Thai patients with optic neuritis.

## 1. Introduction

Optic neuritis is one of the most common optic neuropathies in young patients [1]. Sufferers display decreased vision, color vision deficits, pain on eye movement, and can present with or without a swelling disc appearance. Various forms of this disease, namely optic neuritis-related neuromyelitis optica spectrum disorder (NMOSD-ON), myelin oligodendrocyte glycoprotein (MOG-ON), multiple sclerosis (MS-ON) and double seronegative optic neuritis (DN-ON), with distinct clinical characteristics [1,2,3].

Several factors can affect the visual outcomes of optic neuritis. In a study of Chinese patients [4], females with NMOSD-ON were reported to have achieved good final visual outcomes. Younger age onset has been found to result in a better visual prognosis than older age onset [4,5,6,7]. The prompt administration of intravenous methylprednisolone (IVMP) has been found to improve the chances of favorable visual acuity in both NMOSD- and MOG-related optic neuritis patients [5,7,8], and baseline visual status has been identified as a factor that can affect visual outcomes after treatment [9,10,11]. Regarding the impact of disc morphology on visual outcomes, Ambika et al. stated that patients with a swelling disc appearance at the initial visit had an approximately 4.5-fold better chance of achieving a final visual acuity of greater than 6/18 [12]. Previous studies of Thai populations in the literature have evaluated some predictors affecting visual prognosis using Snellen VA; however, there is limited information on which factors influence visual recovery in terms of improvement in the number of Snellen lines or the logarithm of minimal angle resolution units [10,11].

The purposes of our study were to investigate the clinical characteristics of each subgroup of optic neuritis, and to evaluate the potential factors predicting good visual improvement in Thai patients.

## 2. Materials and Methods

### 2.1. Methods and Data Collection

**Figure 1:**
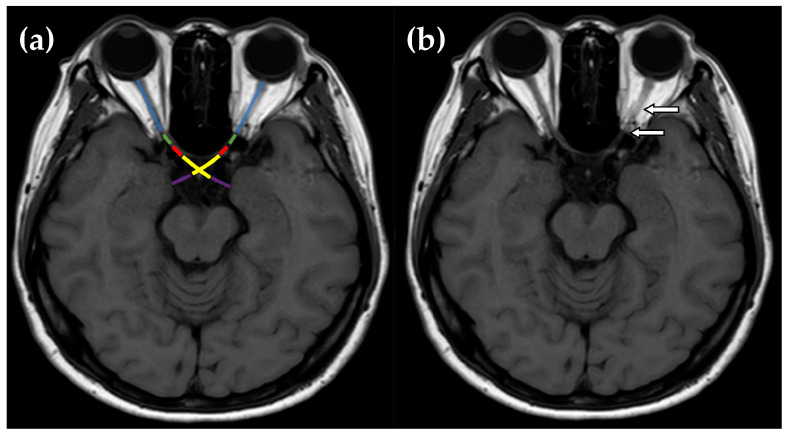
(**a**) Each orbital MRI scan was composed of five segments: orbital optic nerve (blue), intracanalicular optic nerve (green), intracranial optic nerve (red), optic chiasm (yellow), and optic tract (purple). (**b**) MRI T1W scan of a female patient diagnosed with double seronegative optic neuritis showed an orbital and intracanalicular optic nerve enhancement (arrows).

**Figure 2:**
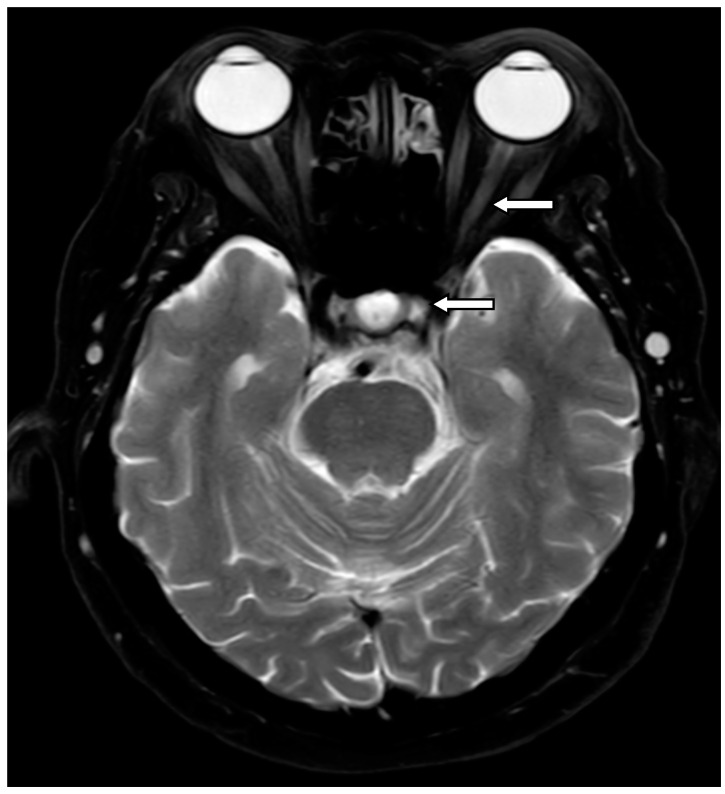
A female patient diagnosed with NMOSD-ON. Her MRI T2W scan showed an enhancing lesion of optic nerve at left intraorbital and optic chiasm (arrows).

The present study included a total of 61 optic neuritis patients who were categorized into 3 groups based on seropositive markers: double seronegative optic neuritis (DN-ON), which was classified in cases of a double seronegative result of aquaporin-4 antibody (Anti-AQP4 Ig-G) and myelin oligodendrocyte glycoprotein antibody (Anti-MOG Ig-G), neuromyelitis optica spectrum disorder-related optic neuritis (NMOSD-ON), and multiple sclerosis-related optic neuritis (MS-ON). Patients’ electrical medical records were searched for the following characteristics: age; gender; baseline clinical characteristics of ophthalmic examinations including corrected visual acuity at nadir and at 12 months, which was converted to logMAR (logarithm of Minimum Angle of Resolution) for statistical analysis; color vision measured by Isihara color plates; disc morphology; presence of relative afferent pupillary defect (RAPD); presence of pain; and laterality. Automated visual field was tested with Humphrey Field Analyzer HFA II 750 using a 30–2 threshold program with the Swedish Interactive Threshold Algorithm (SITA) Fast strategy and reported in decibels (dB). Tests on seromarker were performed in patients with optic neuritis, including AQP4-IgG (using cell-based assay) and MOG-IgG. The number of optic nerve involvements on magnetic resonance imaging (MRI) were divided into 5 segments: intraorbital; intracanalicular; intracranial; optic nerve chiasm; and optic tract [11]. These were then retrospectively reviewed. MRI and segmentation of the optic nerve are schematically shown in Figure 1 and Figure 2. Visual outcome was determined by corrected VA in logMAR between the initial and 12-month follow-up period. In cases of simultaneous optic neuritis, all affected eyes were independently analyzed. For significant improvement in visual outcome, the criterion of improvement of at least 0.3 logMAR has been determined. Stable and worse outcomes are defined when there is no change within the range of −0.3 to +0.3 logMAR, or a decrease of more than −0.3 logMAR at the 12-month follow-up period.

The sole inclusion criterion was diagnosis with optic neuritis between 1 January 2011 and 31 December 2020 by ophthalmologists in our institute. The diagnostic criteria of NMOSD were employed in line with the definition of Wingerchuk et al., and MS was defined in accordance with criteria utilized by McDonald [2,3,13].

Exclusion criteria were the following: (1) other optic neuropathies such as compressive, arteric or non-arteric ischemic optic neuropathy; (2) a follow-up period of less than 12 months; (3) incomplete medical records; and (4) history of prior intravenous methylprednisolone before initial ophthalmic assessments.

### 2.2. Ethical Approval

The present study was approved by Rajavithi Hospital Research Ethics Committee (EC number 011/2565) and adhered to the tenets of the Declaration of Helsinki. All participants gave written informed consent before participating in the study.

### 2.3. Statistical Analysis

All affected eyes were recruited into our study for analysis. Normally distributed continuous data, including patient age and VA converted to logMAR units, were expressed as mean and standard deviation while non-normally distributed data were expressed as median or interquartile range (IQR). Categorical variables (such as gender, presence of RAPD, presence of two consecutive optic nerve involvements on MRI scan) underwent subgroup analysis and chi-square or Fisher exact test. The comparison of continuous data among the three groups of optic neuritis patients employed analysis of variance in normal distribution or Kruskal–Wallis test in non-normal distribution. VA at initial visit was compared with that observed at the end of the 12-month period, and mean deviation (MD) in decibels (dB) of visual field defect was analyzed by paired *t*-test. Univariable and multivariable logistic regression analyses were employed to study factors influencing good visual improvement (at least 0.3 logMAR) at the end of the 12-month period. A *p*-value < 0.2 in univariable analysis was considered to constitute a potential factor and was further investigated with multivariable logistic regression analysis, with a *p*-value of less than 0.05 considered statistically significant. All statistical analyses were performed by SPSS version25 (SPSS Inc., Chicago, IL, USA).

## 3. Results

A total of 140 affected eyes from 130 patients were retrospectively reviewed. Ten patients were found to have simultaneous bilaterality of DN-ON, NMOSD-ON, or MS-ON. In these cases, each affected eye was independently analyzed. We excluded 64 affected eyes as follows: 15 had other optic neuropathies; 30 had a follow-up time of fewer than 12 months; 15 had incomplete medical records; and 4 eyes (of 4 patients) had a history of IVMP therapy before undergoing ophthalmic assessment at our center. A total of 76 affected eyes from 61 patients, therefore, fulfilled our inclusion criteria and were further analyzed in the study.

The 76 affected eyes (of 61 patients) were categorized into three subgroups: 33 patients (40 eyes) were diagnosed as DN-ON (54.1%); 18 (24 eyes) were defined as NMOSD-ON (29.5%); and another 10 (12 eyes) were defined as MS-ON (16.4%). The baseline characteristics of each subgroup are shown in Table 1. The mean ages of those in the DN-ON, NMOSD-ON, and MS-ON groups were 41.2 ± 13.4 years, 46.2 ± 12.6 years, and 28.3 ± 6.6 years, respectively. There was a significant difference between the mean age of patients in the DN-ON and MS-ON groups (*p* = 0.016), and the NMOSD-ON and MS-ON groups (*p* = 0.002); however, no difference was observed between the mean age of those in the DN-ON and NMOSD-ON groups (*p* = 0.505). A female predominance was observed in all three groups, particularly in the NMOSD-ON one, but without any statistically significant difference among the groups (*p* = 0.076). At the initial visit, mean VA at nadir was 0.58 ± 0.39 logMAR, 1.5 ± 0.73 logMAR and 0.84 ± 0.6 logMAR in the DN-ON, NMOSD-ON, and MS-ON groups, respectively. With regard to the baseline visual status in each group, there were significant differences between the MD in dB and baseline VA at nadir in the NMOSD-ON and DN-ON patients, and that of NMOSD-ON and MS-ON patients (all 4 at *p* < 0.001). In the whole cohort, no significant differences were observed among the three groups in terms of the presence of pain, initial disc morphology, percentage of simultaneous bilaterality, relapse or recurrence of optic neuritis, co-existing autoimmune diseases, or duration of onset.

Table 2 displays the MRI characteristics of the three groups of optic neuritis patients at the initial assessment. The majority (40/76) of the affected eyes presented with intraorbital enhancement (37.5% in DN-ON, 70.8% in NMOSD-ON, and 66.7% in MS-ON). There was a significant difference between intraorbital involvement in the DN-ON and NMOSD-ON groups (*p* = 0.01) and also between intracanalicular enhancement in the DN-ON and MS-ON groups (*p* = 0.021). The presence of two consecutive segments of optic nerve lesions was significantly higher in MS-ON patients than in their counterparts in the other two groups (*p* = 0.032).

Table 3 shows the treatment outcomes at the end of the 12-month period for all 76 eyes affected by DN-ON, NMOSD-ON, and MS-ON. The mean 12-month BCVA values in logMAR were 0.26 ± 0.42, 1.23 ± 0.78, and 0.36 ± 0.5, respectively. Notably, there was a significant difference between BCVA in the NMOSD-ON and DN-ON groups, and that of the NMOSD-ON and MS-ON groups (both at *p* < 0.001). No improvement of 0.3 logMAR was observed in NMOSD-ON eyes (0%), and this was significantly different among the three groups (*p* = 0.022). The proportions of color vision improvement and recurrence rates were not significantly different among the three groups (*p* = 0.204, *p* = 0.103, respectively).

Table 4 shows the results after all affected eyes underwent univariable and multivariable logistic regression analysis. DN-ON, NMOSD-ON, a delay in administration of IVMP therapy of greater than 7 days, and poor baseline VA at nadir (VA worse than 20/200) were identified as significant factors in univariable analysis as follows: DN-ON increased the likelihood ratio of attaining an improvement of at least 0.3 logMAR with an odds ratio of 0.16 (95% CI; 0.06–0.449; *p* < 0.001); NMOSD-ON was identified as a significant predictor of the visual outcome with OR = 16.67 (95% CI; 4.763–58.314; *p* < 0.001); a delay of more than 7 days in commencing treatment of IVMP resulted in a higher OR of 2.65 (95% CI; 1.000–7.007; *p* = 0.05); and poor baseline VA at nadir (VA worse than 20/200) increased the risk of poor visual outcomes with an OR of 16.19 (95% CI; 5.097–51.398; *p* < 0.001).

After adjustment of all covariate factors in multivariable logistic analysis, as shown at Table 4, we found that NMOSD-ON was the strongest predictive risk factor of failure to attain visual recovery of at least 0.3 logMAR, with OR = 10.47 (95% CI; 1.095–99.993, *p* = 0.041). A delay in commencing treatment carried an approximately five-fold increase in the risk of poor outcomes with an OR of 5.29 (95% CI; 1.359–20.616, *p* = 0.016). DN-ON and poor baseline VA at nadir failed to reach statistical significance. None of the other variables, including older age onset (≥50 years), female gender, presence of disc swelling, MS-ON, and number of optic nerve involvements of equal or greater than 2 segments on MRI scan were identified as significant predictors in either univariable or multivariable logistic analysis in the present study.

## 4. Discussion

This study of Thai patients revealed that the significant risk factors of having poor visual outcomes were optic neuritis with NMOSD, with an odds ratio of approximately 10 times, and a delay in commencing the administration of intravenous methylprednisolone treatment of more than 7 days, with a five-times higher odds ratio.

Although the optic neuritis treatment trial study (ONTT study) found no convincing evidence of any benefit of prompt IVMP treatment in improving visual acuity at 6 months and 1 year [1], the results of that study may not be suitable for generalization to NMOSD because, as Chen et al. revealed, none of the patients in the ONTT study had NMOSD-ON, and only three cases were diagnosed as MOG-ON [14]. In an experimental study, Dutt et al. stated that there was some merit in this timely treatment when there was inflammation at the optic nerve [15] and the authors concluded that an earlier alleviation of optic neuritis is appropriate in proper disease management. In NMOSD populations, Akashi et al. observed there was an advantage to be gained in administering prompt IVMP in order to achieve improved VA at 1 year [5,16]. Correspondingly, Guo et al. highlighted the better final visual outcomes obtained in NMOSD-ON patients who received this early treatment [4]. Recently, Steibel-Kalish et al. revealed that there was a significant (approximately five times higher) failure rate in the achievement in VA of 20/20, and a ten-fold higher rate of failure to reach VA of 20/30 in cases of a delay in commencing treatment of longer than 7 days in MOG-ON, and in NMOSD-ON at a 3-month visit [7]. In addition, Zhu et al. and Soelberg et al. proposed that there is axonal cell loss within 5–7 days after the onset of optic neuritis [17,18]. Our finding was in line with the results of Stiebel-Kalish et al., revealing that a delay in therapy administration of greater than 7 days significantly increased the risk of poor visual recovery by five times in a multiple logistic model [7].

In this series, NMOSD-ON was detected in 31% of all optic neuritis cases, in agreement with other Asian reports [6,9,10]. Regarding the natural severity of NMOSD-ON, the disease resulted in poor long-term visual outcomes, with a final VA of worse than 20/200 [19,20,21,22], since the histopathology of NMOSD entails autoantibodies attacking the site of AQP4-expression (such as the optic nerve, spinal cord lesions), resulting in astrocytic damage at the optic nerve in patients with optic neuritis. The present study found that the proportions of poor VA at baseline and at 12 months (VA worse than 20/200) were 95.8%, and 58.3%, respectively; moreover, none of the NMOSD-ON patients attained a 0.3 logMAR improvement at the end of the 12-month period. Having NMOSD-ON was identified as the strongest risk factor of poor visual outcome with a ten-fold higher odds ratio in the multivariable logistic model. Our finding was in agreement with another Thai study, which reported that the disease increased the risk of failure to gain a final VA of 20/200 at the final visit [10].

Poor baseline visual acuity at nadir has been identified as a significant predictor of poor outcomes in several studies of both seronegative-ON and NMOSD-ON [10,12,23,24]. However, the current study found that it lost statistical significance in the multivariable logistic model. Likewise, double seronegative optic neuritis also failed to reach statistical strength in our series. We propose as an explanation for this that other studies measured visual outcomes in terms of achievement of improved visual acuity while our study defined this specifically as an improvement of at least 0.3 logMAR, and this could make it difficult to draw a genuine comparison with the results of other research.

With regard to NMOSD-ON, previous researchers have reported poor treatment outcomes for older age onset [4,5,6,7], and this is supported by the theory of Collongues et al. that there is a significant negative association between the healing ability of the central nervous system in patients with NMOSD and older age onset [25]. Regarding the impact of ethnicity on the severity of NMOSD-ON, there are varying conclusions about its association. Kitley et al. found a more severe visual impairment in a UK cohort compared to Japanese NMOSD patients [26]. However, Vanikieti et al. observed no differences in clinical characteristics or visual prognosis between Thai patients and American-Caucasian patients, with the exception of a higher proportion of Thai patients with an initial VA worse than 20/200 [27]. Unfortunately, the participants in this study were all of Thai ethnicity, which limits the examination of the racial aspect in the series. Regarding the age of onset of MS-ON or DN-ON and visual outcomes, Wang et al. studied visual outcomes in people in the age range 45–65 years and those older than 65 years [28]. The authors concluded that there was a significantly better baseline visual status in the younger group; however, final visual acuity was not significantly different in the two age groups of idiopathic, MS-, and NMOSD-related optic neuritis. Similarly, other Asian studies found no correlation between age and final visual outcome [9,10]. In this series, our findings were in agreement with those of several previous reports [9,10,28], revealing no association between visual improvement and age of disease onset; however, these findings should be treated with caution, as there are some points that need to be taken into consideration. First, we set different treatment outcomes from those of other studies, taking visual improvement at 12 months of at least 0.3 logMAR as the criterion for treatment outcome, in contrast to previous articles, which have generally considered the results in terms of final Snellen VA [9,10]. Second, due to the small numbers of each type of optic neuritis, we were not able to test the hypothesis of the association between age in subtypes of optic neuritis and visual outcome, which may introduce some statistical bias. For these reasons, we propose that a large number of participants in each optic neuritis group is essential to provide more evidence in this area of our population.

The present study noted a female predominance, particularly in NMOSD and double seronegative optic neuritis, which is in line with the findings of other reports [4,10,29,30]. Previously, Hussain et al. hypothesized that testosterone could stimulate myelin repair [31]. In addition, Kim et al. observed, in a study of a Korean population [23], that males with NMOSD were less prone than females to suffer from optic neuritis. The impact of gender on visual outcome seems to vary substantially in the literature. Guo et al. proposed that females with NMOSD-ON attained a significantly better therapeutic response [4]; however, some researchers have found contrary results, reporting that there was no association between gender and visual outcome among NMOSD, MS or double seronegative optic neuritis, which was in concordance with our conclusion that gender had no bearing on visual improvement in the three optic neuritis groups [9,10,12].

Currently, the impact of disc morphology on visual outcome is uncertain. Ishikawa et al. found better final vision in NMOSD-ON with disc swelling at the initial visit [30]; however, its statistical significance was lost at the final regression model in predicting the treatment outcomes of all categories (including MOG-ON, NMOSD-ON, and DN-ON). With regard to Asian populations, an Indian study noted that having initial disc swelling at presentation yielded a 4.5 times higher odds ratio of attaining a final VA of greater than 6/18 [12]. Some researchers, however [9,10,30], have contradicted this result, concluding that disc morphology had no impact on visual outcome. Our study was in agreement with the latter researchers, confirming that disc morphology was not a predictor of visual improvement in our series.

Denis et al., employing three-dimensional double inversion recovery of MRI scan (3D-MRI), proposed that optic nerve length involvement is a predictor of retinal neuro-axonal loss and chronic impairment of vision in MS-ON and NMOSD-ON [32]. In addition, Mealy et al. reported that a cut point of optic nerve length involvement of greater than 17.6 mm (mm) from posterior globe to optic nerve pathway was found in NMOSD, whereas optic neuritis with MS presented a shorter length involvement [33]. In a study of Asians, Akashi et al. revealed that the severity of optic nerve involvement determined by the number of optic nerve segments, particularly intraorbital and intracanalicular, in patients with NMOSD-ON was strongly significantly correlated with poor visual outcome [16]. However, a recent Japanese multicenter study reported no association between MRI parameters and final visual acuity in NMOSD, MOG, or double seronegative optic neuritis [30]. In the present study, we found a small number of NMOSD-ON affected eyes presenting with greater than two consecutive segment involvements (16.7%), and this may explain why the number of MRI parameters did not predict visual outcome in our series. We proposed that small numbers of affected eyes may dilute the power of statistical analysis.

Some limitations in the present study should be noted. First, because of its retrospective nature and inevitable missing data, the improvement in some visual parameters such as the change in optic nerve thickness measured by optical coherence tomography (OCT) could not be evaluated as a treatment outcome. Second, our results may not be generalizable for MOG-related optic neuritis groups because our hospital is an adult referral center; therefore, our results may not be applicable in pediatric populations. In addition, our institute treats very few cases of MOG. However, our study reveals that NMOSD-ON carries a risk of poor visual improvement, and it identifies the benefits of rapid treatment of IVMP within 7 days in attaining good visual recovery in Thai patients.

## 5. Conclusions

Neuromyelitis optica spectrum disorder-related optic neuritis (NMOSD-ON) was the second most common cause of optic neuritis and also a predictor of worse visual outcome in our series, and the administration of intravenous methylprednisolone within 7 days may be essential for the attainment of at least 0.3 logMAR of visual recovery in Thai patients.

## Figures and Tables

**Table 1:** Baseline characteristics of DN-ON, NMOSD-ON, and MS-ON patients at initial diagnosis.

Characteristic	Total Eyes (*n* = 76)	DN-ON (*n* = 40)	NMOSD-ON (*n* = 24)	MS-ON (*n* = 12)	*p*-Value	Subgroup Analysis (*p*-Value)		
						DN-ON vs. NMOSD-ON	DN-ON vs. MS-ON	NMOSD-ON vs. MS-ON
Number of patients	61 (100)	33 (54.1)	18 (29.5)	10 (16.4)				
Number of affected eyes	76 (100)	40 (52.6)	24 (31.6)	12 (15.8)				
Mean Age (SD)—yr	40.6 (13.5)	41.2 (13.4)	46.2 (12.6)	28.3 (6.6)	0.002	0.505	0.016	0.002
Female sex—no.	50 (82.0)	27 (81.8)	17 (94.4)	6 (60.0)	0.076	0.209	0.153	0.041
Laterality of first ON presentation—no. (%) Eyes	43 (56.6)	23 (57.5)	14 (58.3)	6 (50.0)	0.88	0.579	0.447	0.451
Bilateral simultaneous	12 (19.7)	6 (18.2)	5 (27.8)	1 (10.0)	0.5	0.325	0.476	0.277
Pain on eye movement—no. (%)	25 (32.9)	10 (25.0)	9 (37.5)	6 (50.0)	0.229	0.218	0.101	0.358
Median duration of onset (SD)—dys	12.3 (13.4)	9.8 (8.7)	15.6 (20.4)	14.8 (9.7)	0.146	0.866	0.042	0.175
Duration > 7 dys (%)	21 (34.4)	11 (33.3)	5 (27.8)	5 (50.0)	0.486	0.468	0.277	0.221
Co-existing autoimmune disease—no. (%)	11 (18.0)	4 (12.1)	6 (33.3)	1 (10.0)	0.131	0.075	0.672	0.184
Systemic lupus erythematosus	9 (14.7)	4 (12.1)	4 (22.3)	1 (10.0)	0.56	0.287	0.672	0.389
Graves’ disease	2 (3.2)	0 (0)	2 (11.2)	0 (0)	0.085	0.12	-	0.405
Visual parameter—no. (%)								
VA logMAR at nadir (SD)	0.91 (0.68)	0.58 (0.39)	1.5 (0.73)	0.84 (0.6)	<0.001	<0.001	0.432	0.003
VA Snellen at nadir (%)					<0.001	<0.001	0.229	<0.001
VA 20/20–20/60	13 (17.1)	11 (27.5)	0 (0)	2 (16.7)				
VA 20/70–20/200	34 (44.7)	26 (65.0)	1 (4.2)	7 (58.3)				
Poor baseline VA (20/200)	29 (38.2)	3 (7.5)	23 (95.8)	3 (25.0)				
Visual field at nadir (dB)	15.4 (9.95)	10.6 (7.1)	25.3 (7.1)	11.6 (8.9)	< 0.001	<0.001	0.871	<0.001
Abnormal color vision (%)	49 (64.5)	27 (67.5)	16 (66.7)	6 (50.0)	0.035	0.008	0.365	0.038
Presence of swollen optic disc (%)	37 (48.7)	18 (45.0)	12 (50.0)	7 (58.3)	0.711	0.448	0.315	0.454
Relapse rate (%)	1 (1.6)	0 (0)	1 (5.6)	0 (0)	0.297	0.353	-	0.643
Recurrent—no. (%)	2 (3.3)	1 (3.0)	0 (0)	1 (10.0)	0.36	0.647	0.415	0.357

DN-ON, NMOSD-ON, MS-ON, and LogMAR denote double seronegative optic neuritis, neuromyelitis optica spectrum disorder optic neuritis, multiple sclerosis optic neuritis and Logarithm of Minimum Angle of Resolution, respectively. Individual patient data were used to analyze variables such as female gender, bilateral simultaneous, mean duration of onset, duration > 7 days, co-existing autoimmune disease relapse, and recurrence. *p* values of 0.05 were considered significant, and *p* values of 0.025 were considered significant in the subgroup analysis. The following is the VA Snellen range: VA 20/20–20/60, VA 20/70–20/200 and >20/200. SD stands for standard deviation, and the visual field is measured in decibels (dB). A poor baseline VA was defined as an initial VA of worse than 20/200. In the variables mean duration of onset and visual field at nadir, the Kruskal–Wallis test was used. Relapse and recurrent are defined as repeated attacks of optic neuritis in the same eye within 30 days and more than 30 days retrospectively.

**Table 2:** MRI characteristics at the initial assessment of DN-ON, NMOSD-ON, and MS-ON of each affected eye.

Characteristic	Total Eyes (*n* = 76)	DN-ON (*n* = 40)	NMOSD-ON (*n* = 24)	MS-ON (*n* = 12)	*p*-Value	Subgroup Analysis (*p*-Value)		
						DN-ON vs. NMOSD-ON	DN-ON vs. MS-ON	NMOSD-ON vs. MS-ON
Segmental enhancement of AVP—no. (%)								
Orbital optic nerve	40 (52.6)	15 (37.5)	17 (70.8)	8 (66.7)	0.02	0.01	0.073	0.544
Intracanalicular optic nerve	10 (13.2)	2 (5.0)	4 (16.7)	4 (33.3)	0.032	0.135	0.021	0.236
Intracranial optic nerve	6 (7.9)	1 (2.5)	0 (0)	1 (8.3)	0.126	0.061	0.412	0.451
Optic chiasm	0 (0)	0 (0)	0 (0)	0 (0)	-	-	-	-
Optic tract	0 (0)	0 (0)	0 (0)	0 (0)	-	-	-	-
≥2 consecutive segments	10 (13.2)	2 (5.0)	4 (16.7)	4 (33.3)	0.032	0.135	0.021	0.236

DN-ON, NMOSD-ON, and MS-ON denote double seronegative optic neuritis, neuromyelitis optica spectrum disorder optic neuritis, and multiple sclerosis optic neuritis. AVP denotes anterior visual pathway. *p* values of 0.05 were considered significant, and *p* values of 0.025 were considered significant in the subgroup analysis.

**Table 3:** Treatment outcomes of DN-ON, NMOSD-ON, and MS-ON of each affected eye (12 months).

Characteristic	Total Eyes (*n* = 76)	DN-ON (*n* = 40)	NMOSD-ON (*n* = 24)	MS-ON (*n* = 12)	*p*-Value	Subgroup Analysis (*p*-Value)		
						DN-ON vs. NMOSD-ON	DN-ON vs. MS-ON	NMOSD-ON vs. MS-ON
Number of patients	61 (100)	33 (54.1)	18 (29.5)	10 (16.4)				
Final BCVA—no. (%)								
LogMAR—mean ± SD	0.58 ± 0.72	0.26 ± 0.42	1.23 ± 0.78	0.36 ± 0.5	<0.001	<0.001	0.771	<0.001
VA 20/20–20/60	46 (60.5)	35 (87.5)	2 (8.3)	9 (75.0)	<0.001	<0.001	0.561	<0.001
VA 20/70–20/200	11 (14.5)	2 (5.0)	8 (33.3)	1 (8.3)				
VA < 20/200	19 (25.0)	3 (7.5)	14 (58.3)	2 (16.7)				
Mean difference of final VA vs. at nadir	0.33 ± 0.34	0.32 ± 0.23	0.27 ± 0.38	0.48 ± 0.53	0.002	0.004	0.131	0.006
Visual acuity outcome—no. (%)					0.022	0.092	0.176	0.003
Improve (≥0.3 LogMAR)	2 (2.6)	1 (2.5)	0 (0)	1 (8.3)	-	-	-	-
Stable	50 (65.8)	25 (62.5)	21 (87.5)	4 (33.3)	-	-	-	-
Worse (≤−0.3 LogMAR)	24 (31.6)	14 (35.0)	3 (12.5)	7 (58.3)	-	-	-	-
Color vision improvement (%)	29 (38.2)	19 (47.5)	7 (29.2)	3 (25.0)	0.204	0.118	0.147	0.56
Recurrent—no. (%)	3 (2.6)	1 (2.5)	0 (0)	2 (16.6)	0.103	0.625	0.129	0.105

DN-ON, NMOSD-ON, and MS-ON denote double seronegative optic neuritis, neuromyelitis optica spectrum disorder optic neuritis, and multiple sclerosis optic neuritis. *p*-values of 0.05 were considered significant, and *p*-values of 0.025 were considered significant in the subgroup analysis. Improvement, stability, and deterioration of vision were classified at 12 months compared to at nadir as follows: ≥0.3 LogMAR, between >−0.3 and <0.3 LogMAR and ≤−0.3 LogMAR retrospectively. Recurrence was defined as repeated attacks of optic neuritis in the same eye with an interval between the attacks of more than 30 days.

**Table 4:** Logistic-regression analysis of factors predicting visual outcome at 12-months.

	OR	95% CI	*p*-Value
Univariable model			
Age ≥ 50 yrs	1.33	0.468–3.781	0.59
Female gender	0.96	0.298–3.111	0.95
Duration ≥ 7 dys	2.65	1.000–7.007	0.05
Disc swelling	1.69	0.675–4.239	0.262
MRI ≥ 2 segments	2.31	0.593–8.974	0.227
DN-ON	0.16	0.060–0.449	<0.001
NMOSD-ON	16.67	4.763–58.314	<0.001
MS-ON	0.4	0.100–1.625	0.201
Poor VA at nadir	16.19	5.097–51.398	<0.001
Multivariable model			
Duration ≥ 7 dys	5.29	1.359–20.616	0.016
DN-ON	1.4	0.236–8.305	0.711
NMOSD-ON	10.47	1.095–99.993	0.041
Poor VA at nadir	4.7	0.767–28.78	0.094

DN-ON, NMOSD-ON, MS-ON, and MRI denote double seronegative optic neuritis, neuromyelitis optica spectrum disorder optic neuritis, multiple sclerosis optic neuritis and magnetic resonance imaging. A poor baseline VA was defined as an initial VA of worse than 20/200. ≥2 MRI segments consecutively. OR and CI denote odds ratios and confidence interval. *p* values of 0.05 were considered significant.

## Data Availability

The datasets used and/or analyzed during the current study are available from the corresponding author upon reasonable request.
